# Teaching the internist to see: effectiveness of a 1-day workshop in bedside ultrasound for internal medicine residents

**DOI:** 10.1186/s13089-016-0047-7

**Published:** 2016-08-11

**Authors:** Ryan D. Clay, Elizabeth C. Lee, Marc F. Kurtzman, Renee K. Dversdal

**Affiliations:** 1 Department of Medicine, Oregon Health & Science University, Portland, USA; 2 Division of Hospital Medicine, Department of Medicine, Oregon Health & Science University, 3181 SW Sam Jackson Park Road, OP-30, Portland, OR 97239 USA; 3 Mayo Clinic Minnesota, 200 First Street SW, Rochester, MN USA

**Keywords:** Bedside ultrasound, Physical examination, Point-of-care ultrasound, Graduate medical education, Ultrasound education, Internal Medicine education

## Abstract

**Background:**

A growing body of evidence supports the use of bedside ultrasound for core Internal Medicine procedures and increasingly as augmentation of the physical exam. The literature also supports that trainees, both medical students and residents, can acquire these skills. However, there is no consensus on training approach.

**Aim:**

To implement and study the effectiveness of a high-yield and expedited curriculum to train internal medicine interns to use bedside ultrasound for physical examination and procedures.

**Setting:**

The study was conducted at a metropolitan, academic medical center and included 33 Internal Medicine interns.

**Program description:**

This was a prospective cohort study of a new educational intervention consisting of a single-day intensive bedside ultrasound workshop followed by two optional hour-long workshops later in the year. The investigation was conducted at Oregon Health & Science University in Portland, Oregon. The intensive day consisted of alternating didactic sessions with small group hands-on ultrasound practice sessions and ultrasound simulations. A 30-question assessment was used to assess ultrasound interpretation knowledge prior to, immediately post, and 6 months post intervention.

**Results:**

Thirty-three interns served as their own historical controls. Assessment performance significantly increased after the intervention from a mean pre-test score of 18.3 (60.9 % correct) to a mean post-test score 25.5 (85.0 % correct), *P* value of <0.0001. This performance remained significantly better at 6 months with a mean score of 23.8 (79.3 % correct), *P* value <0.0001. There was significant knowledge attrition compared to the immediate post-assessment, *P* value 0.0099.

**Conclusions:**

A single-day ultrasound training session followed by two optional noon conference sessions yielded significantly improved ultrasound interpretation skills in internal medicine interns.

## Background

The twenty-first century has witnessed a dramatic and rapid expansion of the availability and use of technology in patient care. Improvement of pre-existing technologies, such as the miniaturization of ultrasound devices, has significantly expanded the range of use for these devices. Trauma Surgery pioneered the use of point-of-care ultrasound. Emergency Medicine followed by Critical Care then embraced and popularized bedside, provider-performed ultrasound, beginning in the 1970s. Many bedside ultrasound applications have been validated, and several specialties have ACGME training requirements for training in ultrasound. The Internal Medicine community has been slower to incorporate bedside ultrasound into daily practice. However, the literature examining the use of bedside ultrasound by generalists and hospitalists for diagnostic and procedural purposes is now growing.

Now that the value of ultrasound use is increasingly recognized by Internal Medicine providers, focus has also expanded to ultrasound education in residency training. In 2012, approximately 25 % of Internal Medicine residency programs had some form of ultrasound training with another 25 % planning on incorporating ultrasound training in the next year [1]. Additionally, there is an increasing body of literature describing the efficacy of curricular modules to teach ultrasound skills to Internal Medicine residents [2]. Programs using both cadaver models and standardized patients have demonstrated improvement in resident ultrasound examination skills after a hands-on training module [3, 4]. Other approaches include a 30-h ultrasound course covering many applications held during intern orientation, and an integrated three-year curriculum focused on the cardiovascular limited ultrasound examination (CLUE) [2, 5]. When these ultrasonography skills are applied to clinical care, patients may receive a more accurate or nuanced diagnosis, expediting their appropriate workup and treatment [6].

In addition, residents also gain confidence from formal training in using ultrasound for invasive procedures [7]. Studies demonstrate that ultrasound-guidance improves the safety of central line placement and thoracentesis [8, 9]. There is evidence that the patients of residents trained in bedside ultrasound-guided paracentesis not only received less post-procedural blood product transfusions compared to patients that underwent paracentesis by Interventional Radiology, but also benefitted from an average total cost reduction of over $500 per procedure for bedside ultrasound-guided paracentesis [10]. This is potentially related to Interventional Radiology versus American Association for the Study of Liver Diseases consensus guidelines regarding ideal INR and platelet count before paracentesis, and is an important consideration in the ever-important concept of High Value Care.

In order to foster career-long proficiency with ultrasound technology, it was deemed important to introduce skills training early in Internal Medicine residency. However, the optimal timing and intervention to fit high-yield ultrasound training into a crowded curriculum with limited resources is not clear. The Oregon Health and Sciences University Internal Medicine residency program is located at a metropolitan academic medical center in Portland, Oregon, and includes 33 interns. This intervention focused on a single-day bedside ultrasound training followed by two optional 1-h-long workshops later in the year. It was hypothesized that after 1 day of intensive ultrasound training interns would demonstrate significantly improved ultrasound interpretation skills, along with significant retention at 6 months.

## Methods

This was a prospective cohort study with 33 interns serving as their own historical controls. A 1-day bedside ultrasound workshop was developed as one component of a comprehensive 5-day intern “boot-camp” designed to improve interns’ cognitive, affective and psychomotor skills using active learning techniques including simulation, standardized patients and communication workshops. The “boot-camp” was purposefully placed 3 months into the academic year so that these key concepts did not get lost during the intern’s busy orientation time.

The bedside ultrasound training day consisted of alternating didactics with small group hands-on sessions. Six topics pertinent to Internal Medicine patient management were covered including ultrasound basics (physics, knobology, artifacts), cardiac limited ultrasound exam (CLUE) [5], basic pulmonary exam, abdominal exam, basic vascular anatomy of the aorta, and neck anatomy. The abdominal exam was a modified FAST exam adapted for internal medicine, assessing for free intraperitoneal fluid, inferior vena cava collapse as an assessment of intravascular volume status [11], and ruling out hydronephrosis. Each topic was introduced with a 15- to 20-min didactic. Following the brief didactic session, interns worked within 12 pre-assigned groups of 2–3 learners for 40-min hands-on sessions. Each session was divided into 20 min for completing modules demonstrating pathology on SonoSim^®^ machines and 20 min for facilitator-led hands-on practice with a SonoSite Edge^®^ machine and volunteer models. Volunteers were solicited from the pre-health student list serve of a local university. Facilitators included two clinical trainers from SonoSite^®^ in addition to emergency medicine residents, fellows and faculty, cardiology fellows, and Internal Medicine faculty. All facilitators had experience and training in bedside ultrasound. The teaching during the intensive intervention was re-enforced by two additional 1-h ultrasound training sessions within the first 6 months. However, these were held during noon conference sessions with untracked attendance.

**Fig. 1:**
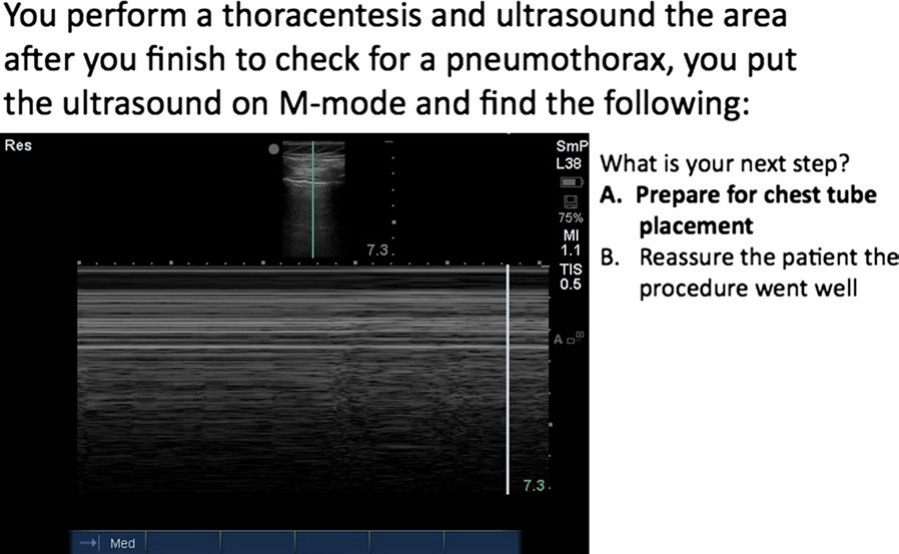
Sample test question of clinical application of bedside ultrasound

To assess image acquisition, optimization, interpretation and application within the clinical context, study investigators designed a 30-question assessment covering ultrasound basics, neck anatomy, abdomen, limited cardiac, volume assessment, and basic vascular ultrasound. Subject areas were determined by group consensus and based on existing literature supporting the use of bedside ultrasound in the practice of internal medicine. Questions were vetted by the four co-investigators with final edits by the PI. Seventeen questions tested image interpretation, 7 tested concepts of image acquisition/optimization and 6 tested clinical application. A copy of the assessment can be obtained by emailing the corresponding author. See Fig. 1 for an example, test question.

Interns completed the ultrasound knowledge assessment in a proctored room directly before and after the bedside ultrasound training day. They subsequently completed the ultrasound knowledge assessment approximately 6 months after the bedside ultrasound training day on their own time in an unproctored setting. Each individual testing session randomized the question order and did not provide feedback regarding test performance to minimize subjects’ ability to “learn the test”.

Qualitative feedback on the entire training week and each session was collected from learners at the completion of the week via survey.

This study was approved by the Oregon Health & Science University Institutional Review Board. All participants in the intern intensive week gave informed consent to be studied as part of this educational initiative; however, the IRB did waive documentation of consent and interns were notified that the research portion was optional. Each intern selected a unique identifier that test results could be traced to at the onset of the study. Intern identifiers were not known to the data assessors, and all participants were assured that their scores were used only for research purposes rather than summative feedback.

Statistical analysis was calculated using Stata v. 11 using Student’s *t* test for mean comparison.

## Results

After assessing for proper matching of the pre- and post-test data sets, as well as verifying individual respondents based upon their unique identifiers, 33 interns completed both the pre and post-assessment. The 6-month post-assessment was completed by 24 of the original respondents. This gave a follow-up response rate of 72.7 %. The average intern age was 28.3 years old; only 29.4 % reported significant prior exposure to ultrasound teaching before residency. A strong majority (88.2 %) anticipated performing procedures as part of their medical practice after graduating from residency (Table 1). Nearly all of the interns had some inpatient experience by the time of the workshop and over half of them had rotated through the medical intensive care unit (Table 1).

**Table 1:** Demographics of interns participating in “intensive week” curriculum

Interns (*n* = 33)	
Average age (years)	28.3
Percent female	66.7
Percent with prior ultrasound training	29.4
Anticipates performing procedures in post residency career? (% yes)	88.2

**Fig. 2:**
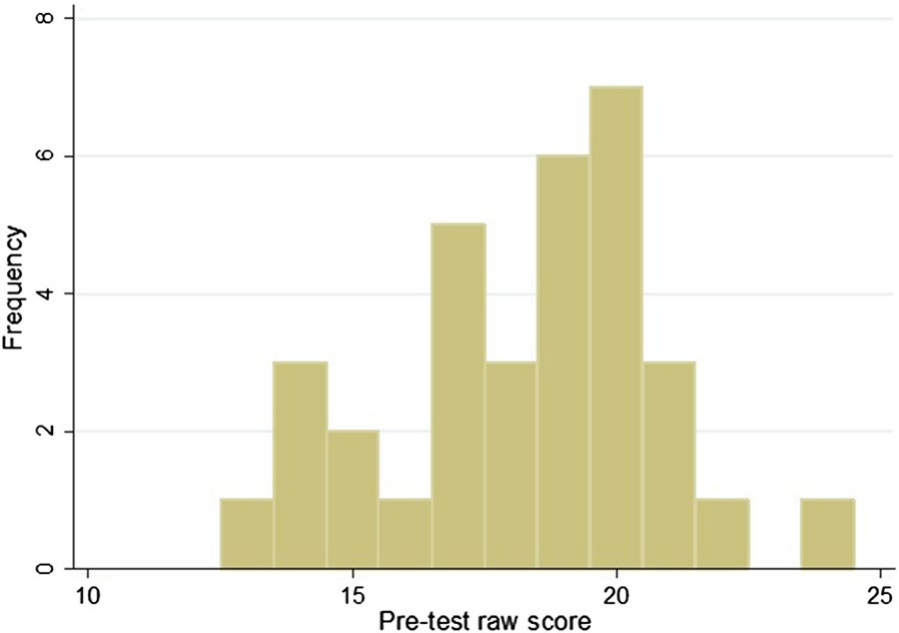
Distribution of pre-test raw scores (of a possible 30 points), *n* = 33

**Fig. 3:**
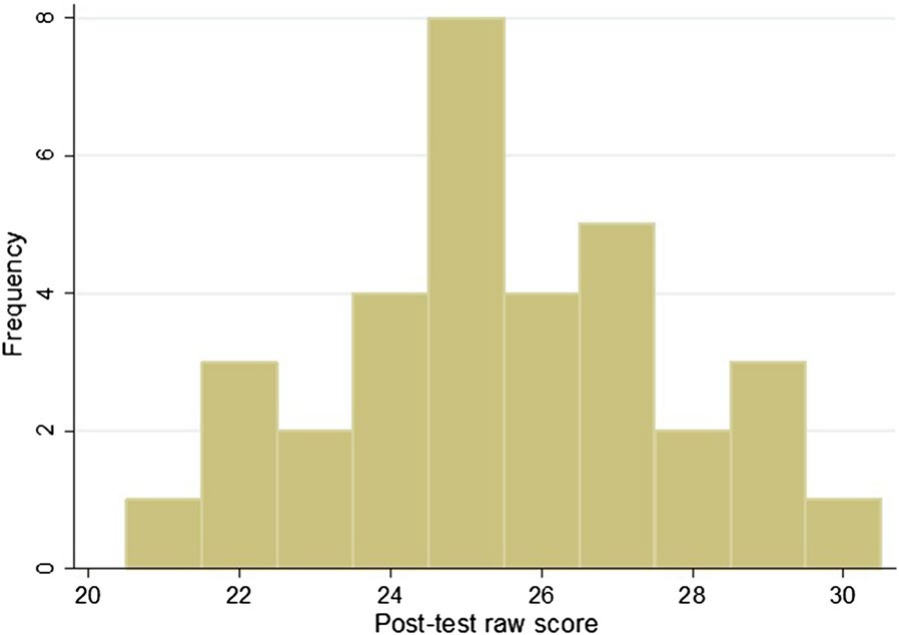
Distribution of immediate post-test raw scores (of a possible 30 points), *n* = 33

**Fig. 4:**
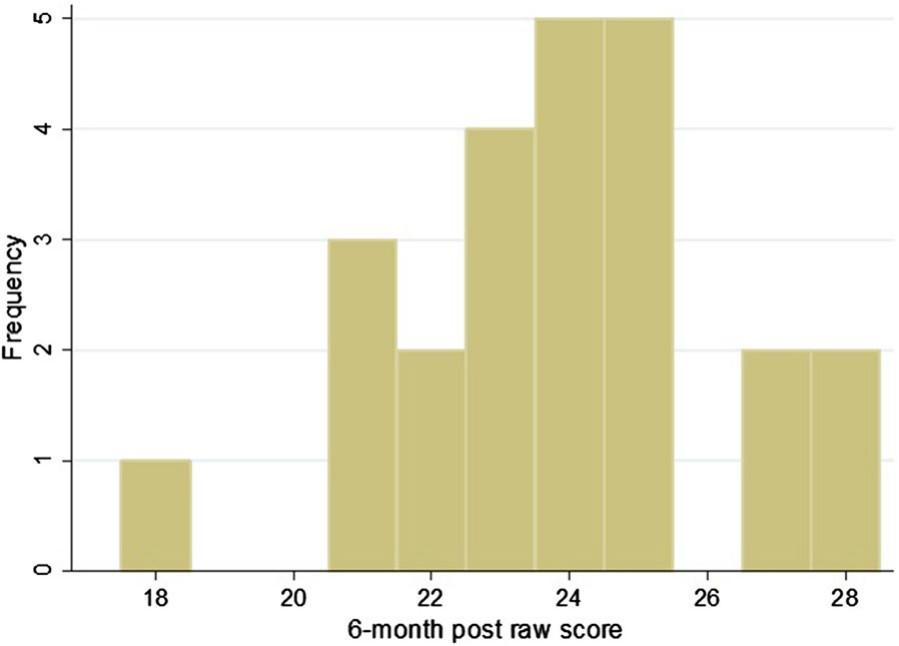
Distribution of 6-month post-test raw scores (of a possible 30 points), *n* = 24

The mean pre-test score was 18.3 (60.9 %, 95 % CI 17.4–19.2) with a distribution as seen in Fig. 2, and the mean post-test score was 25.5 (85.0 %, 95 % CI 24.7–26.3) distrubution demonstrated in Fig. 3. A two-sample paired *t* test demonstrated that the post-test scores were significantly higher with a *P* value of <0.0001. Interns then completed the assessment 6 months after their initial training session. The mean 6-month post-test score was 23.8 (79.3 %), 95 % confidence interval 22.8–24.8, with distrubution demonstrated in Fig. 4. This remained statistically significantly higher than the pre-test score (*P* < 0.0001), but was lower than the immediate post-assessment (*P* = 0.0099).

**Fig. 5:**
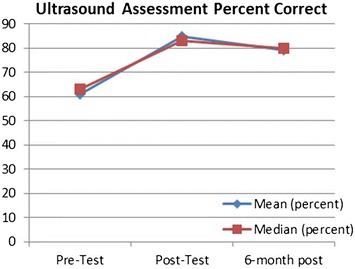
Assessment percent correct—mean and median

Mean and median test scores were closely associated as demonstrated by Fig. 5, and the mean fell within the standard deviation for all assessment periods. Additionally, the histograms reflect a change in the distribution of scores after the teaching intervention and this change was partially maintained 6 months out from the initial intervention.

The study also included a qualitative assessment of the ultrasound intervention based upon intern feedback where each session of the intern boot-camp was ranked on a 1–5 Likert scale. The ultrasound course was rated a mean of 4.4, fitting with an overwhelmingly positive response from the interns. One intern acknowledged that ultrasound is “such a common bedside tool.” Another noted that bedside sonography “is an ongoing skill, but something that will definitely be a part of my future practice.” Participants expressed a desire for more hands-on practice and less didactic time. Learner opinion of ultrasound simulators compared to volunteer subjects was not specifically quantified; however, 2 of the 33 subjects commented on desiring more time scanning the live models as opposed to practice scenarios on the simulator.

## Discussion

These results show that an innovative 1-day bedside ultrasound curriculum timed early in the academic year supplemented by two 1-h workshops results in significant improvement in point-of-care ultrasound knowledge pertaining to image acquisition, optimization, interpretation and clinical application. This knowledge was retained to a statistically significant degree 6 months later, though with some knowledge decay. Additionally, the intervention was very positively received by learners who felt that these skills would serve them well in their future Internal Medicine careers. Timing this intervention as a distinct entity, separate from intern orientation, may have allowed for better focus on ultrasound-specific educational goals.

This study has several limitations. There was significant attrition in the 6-month assessment group (*n* = 7, 27.3 %), which could leave a self-selecting group of ultrasound enthusiasts completing the knowledge assessment. Additionally, the 6-month knowledge assessment was performed in an unsupervised setting, while the immediate pre- and post assessments were conducted in a proctored environment. Finally, this assessment evaluated ultrasound skills and use by multiple-choice testing, and thus actual image acquisition at the bedside could not be tested. However, we carefully crafted questions that referred to image aquisition such as probe selection for a specific exam, optimization techniques such as depth adjustment, and clinical interpretation such as appropriate next step in an attempt to assess more than simple ultrasound interpretation skills.

Strengths of this study include the condensed time frame with minimal time investment in an already overcrowded curriculum, demonstration of knowledge retention that persisted after the immediate post-training assessment, and relative lack of expense to recreate with the use of volunteer faculty instructors and volunteer ultrasound scanning models.

It is clear that a landmark-based approach to procedures is less safe than ultrasound-guided approach for procedures in Internal Medicine. Furthermore bedside sonography adds to the physical exam to offer the opportunity to better understand complex patients early in the course of their illness [12–14]. The majority of intern participants anticipate performing procedures as part of their practice after residency. However, less than 1/3 of the entering class had prior ultrasound teaching, despite bedside ultrasound’s essential role in bedside procedures. As an internal medicine residency training tomorrow’s general internist, we feel it is our duty to include high-quality training in ultrasound for our trainees. But as noted, funding and time constraints raise a considerable challenge to adding new curricula.

Including a module with just 80 min of hands-on practice significantly increased internal medicine residents’ comfort in interpreting and acquiring images [7]. However, the optimal training duration is still unclear, as another program’s experience with a 3-h stand-alone training session did not demonstrate improved diagnostic accuracy [15]. This study shows that a 1-day workshop with two 1-h sessions within the next 6-month results in sustained knowledge of image acquisition theory and image interpretation as well as improved attitudes towards the use of bedside ultrasound. At a time when Medicare funding for post-graduate medical education stagnates, it is ever more important to implement cost-effective educational interventions while expanding the breath of Internal Medicine curricula to embrace technologic advances in patient care.

## Conclusion

While there are many ways to introduce and teach ultrasound skills to learners, timing and funding constraints may pose a barrier to adopting new curricula. In this study, a focused one-day intensive curriculum with two supplemental 1-h workshops yielded improved ultrasound interpretation, troubleshooting and clinical application knowledge immediately afterwards and at 6-month post-training. Future studies are needed to formally assess ultrasound image acquisition skills, and clinical outcomes related to the addition of bedside ultrasound to the standard clinical assessment by Internal Medicine physicians.

## Abbreviations

ACGME: Accrediting Committee of Graduate Medical Education
CLUE: Cardiovascular limited ultrasound examination
FAST: Focused assessment with sonography in Trauma
